# Sex Differences in the Pathophysiology, Treatment, and Outcomes in IHD

**DOI:** 10.1007/s11883-015-0511-z

**Published:** 2015-05-03

**Authors:** Monika Sanghavi, Martha Gulati

**Affiliations:** Department of Internal Medicine, Division of Cardiology, University of Texas Southwestern Medical Center, 5323 Harry Hines Blvd, Dallas, TX 75390-8830 USA; Department of Internal Medicine (Cardiology) and Department of Clinical Public Health (Epidemiology), The Ohio State University, Columbus, OH USA

**Keywords:** Ischemic heart disease, Women, Microvascular dysfunction, Cardiovascular disease, Risk factors

## Abstract

Heart disease is the number one killer of women. Although there are many similarities between men and women, the evolving understanding of ischemic heart disease in women allow us to emphasize the important differences that need to be recognized. These differences, including symptoms at presentation, importance of particular risk factors, pathophysiology of disease, and treatments/outcomes, will be discussed in this review.

## Introduction

Women in the USA are more likely to die of cardiovascular disease (CVD) than any other cause [1••]. In fact, cardiovascular disease claims the life of a woman every minute [1••]. The statistics are staggering; however, almost 50 % of white women and three fourths of Hispanic and black women are still unaware that this is their greatest risk [2].

Among cardiovascular diseases, coronary heart disease (CHD) makes up the majority of events for both men and women below 75 years of age [1••]. The incidence of CHD in women lags behind men by 10 years, suggesting a protective effect in women that is lost with advanced age, particularly after the onset of menopause. The overall incidence of CHD is lower in women; however, across all age strata, a myocardial infarction (MI) is more likely to be fatal in women, particularly in younger women (under 55 years of age) [1••]. Although the overall trends suggest a decrease in incident CHD events and CHD-related deaths over the past 20–25 years in both men and women, the only exception is younger women (35–44 years of age) for whom the mortality has increased [3].

Previously it was assumed that heart disease in women was the same as in men, and the underrepresentation of women in research studies prevented any alternate sex-specific conclusions. With the emergence of new sex-specific studies and data, the landscape of heart disease is changing. We now know that certain risk factors are stronger predictors of heart disease in women, there are sex differences in symptoms, and there are differences in the underlying pathophysiology. With the new understanding of the pathophysiologic differences come changes in diagnostic testing and treatment strategies.

CHD is traditionally characterized by obstructive atherosclerosis in the epicardial coronary arteries resulting in ischemia or decreased myocardial blood flow. However, with the recognition that there are a variety of disorders that result in ischemia and ischemic symptoms in women, not just coronary heart disease, the more-inclusive term ischemic heart disease (IHD) is considered fitting for this discussion [4]. IHD is a broader term that encompasses any disorder or disease that results in myocardial ischemia; this includes Cardiac Syndrome X, a term used to describe patients with symptoms and evidence of ischemia but no obstructive coronary artery disease [5] and is noted to be more common in women. More recently, this syndrome has been labeled as female-specific IHD.

The following review article will discuss the sex differences in IHD with a focus on the pathophysiology, treatment, and outcomes.

## Symptoms/Clinical Presentation

Angina pectoris is the most common symptom of myocardial ischemia. The description of “typical” angina was based the presence of classic characteristics of obstructive coronary disease which included retrosternal chest pressure, exacerbation with activity, and relief with rest or nitroglycerin, defined in a population of predominantly men. Of note, other causes of myocardial ischemia may not present with the same characteristic presentation, for example, patients with coronary vasospasm often report pain at rest. The distinction of “typical” vs. “atypical” angina, based on the number of classic characteristics present, was used by Diamond and Forrester to help determine the pretest probability of atherosclerotic disease [6]. More recently, the term “atypical angina” is often used when describing symptoms in women since some women can have prodromal symptoms of shortness of breath, fatigue, and weakness with ischemia [7] or other nonclassic descriptions of pain. However, even though women are more likely to have atypical symptoms when compared to men, the most common presentation in acute coronary syndrome is still typical angina [8]. In a study evaluating anginal symptoms in men and women with confirmed obstructive coronary artery disease, there was no difference in the presenting symptoms [9]. In general, women have a higher prevalence of angina than men [10] and more functional impairment from the pain. Interestingly, even when typical angina is present, women are less likely to have any evidence of obstructive CAD when angiography is performed [11]. In women with signs and symptoms of IHD yet without obstructive CAD, the majority will have repeated episodes of chest pain requiring hospitalization and repeat testing which is economically taxing on the healthcare system [12].

## Risk Factors

### Traditional Risk Factors

The absence of traditional cardiac risk factors in midlife is associated with a low lifetime risk of heart disease [13•]. However, by the age of 55, a majority of women have at least one major risk factor putting them at an increased lifetime risk of cardiovascular disease [13•]. Traditional cardiac risk factors play a key role in the development of heart disease in women and men, but the prevalence of certain risk factors differ between the sexes and some are stronger predictors of heart disease in women. Women have lower total cholesterol levels than men until after the fifth decade of life, thereafter their values are greater [14]. In general, high-density lipoprotein cholesterol (HDL-C) levels are higher in women than in men, but decrease during the menopause transition likely due to hormonal changes [15]. In addition to dyslipidemia, postmenopausal women also have a clustering of other risk factors including obesity and hypertension that could be related to gender-specific metabolic differences exacerbated by hormonal imbalances [14]. Diabetes, metabolic syndrome, hypertension, obesity, and hypertriglyceridemia are all stronger risk factors for ischemic heart disease in women than men [16–20]. A recent meta-analysis reported that women with diabetes had a 40 % greater risk of incident CHD compared with men with diabetes [17]. In fact, the presence of diabetes is thought to take away the relative protection from ischemic heart disease in young women [14].

Even though there is a lower prevalence of smoking among women than men (15.9 % vs. 20.5 %) (1), smoking confers a higher risk of ischemic heart disease in women. In fact, for a woman, the risk of CHD mortality from cigarettes is equivalent to the risk associated with weighing ~42 kg more than her nonsmoker counterpart [14]. A recent meta-analysis demonstrated a 25 % higher relative risk of heart disease in women smokers compared to men [21].

There is increasing recognition of the importance of lifestyle on ischemic heart disease. In a recent study evaluating young women without underlying CVD disease or CV risk factors, it was noted that 73 % of CHD cases and 46 % of CVD risk factor cases were attributable to a poor lifestyle [22••]. This emphasizes the importance of primordial prevention with education and lifestyle counseling at a young age.

Physical activity and inactivity are both important considerations. A recent study found that after the age of 30, the population risk of heart disease attributable to physical inactivity outweighed all other risk factors in women [23]. On the other end of the spectrum, exercise capacity has a strong and independent positive association with cardiovascular and all-cause mortality [24]. The adjusted hazard ratio for every 1-MET decrement in exercise capacity is 1.20 [25].

### Nontraditional Risk Factors

Psychosocial factors are known to be associated with an increased incidence of IHD as well as recurrent CV events in patients with established disease [26] and can prevent individuals from adopting recommended lifestyle changes [27]. Psychosocial problems such as depression are twice as common in women than in men [28]. For women, family conflicts and obligations, depression and anxiety are all associated with increased risk of heart disease whereas for men, work obligations and hostility are more commonly associated with IHD risk [29]. The differential impact of mental stress on women compared to men was demonstrated in a recent study in post-MI patients aged 38–60 years, younger women (those less than 50 years of age) had increased evidence of mental stress-induced myocardial ischemia as determined by the sum difference score (SDS) on technetium-99 m perfusion imaging when compared to young men. There was no difference seen with exercise-induced ischemia or in the older subjects (those >50 years of age) [30].

Inflammatory markers such as high-sensitivity C-reactive protein (hs-CRP) can be used in addition to traditional cardiac risk factors for further risk stratification in men and women. An elevation is associated with a greater risk of IHD even when accounting for traditional risk factors. High-sensitivity CRP is consistently higher after puberty in women [31] and has been shown to vary with levels of estrogen in postmenopausal women [32]. It has also been demonstrated to help stratify higher risk women with metabolic syndrome. In one study of women with metabolic syndrome [33], those with hs-CRP >3.0 mg/l had twice the risk of future cardiovascular events compared to those with hs-CRP <3.0 mg/l.

Autoimmune disorders characterized by chronic inflammation such as systemic lupus erythematosus (SLE) and rheumatoid arthritis (RA) primarily affect women and support the theory that inflammation may be associated with atherosclerosis. These individuals often manifest ischemic heart disease at an earlier age and have a rapid progression of atherosclerosis [34]. Young women with SLE are 50 times more likely to have an acute MI than women of the same age without it [35].

### Sex-Specific Risk Factors

As mentioned earlier, women have a 10-year relative delay in the clinical expression of IHD that is not completely understood. Clinicians suspect that the delay is due to the protective effects of estrogen during a woman’s reproductive years, since estrogen has anti-atherosclerotic and anti-inflammatory effects [36], as well beneficial effects on lipids and endothelial vasomotor function [37]. However, despite making physiologic sense, hormone replacement therapy as a preventive measure for women has not proven to be effective for primary or secondary prevention of cardiovascular disease [38, 39].

There is increasing recognition of pregnancy-related complications such as preeclampsia, gestational diabetes, and gestational hypertension as being risk factors for ischemic heart disease. In fact, women with a history of preeclampsia have twice the risk of cardiovascular disease and venous thromboembolism in the decade following their pregnancy [40] as well as an increased risk of chronic kidney disease and diabetes mellitus [41]. Recently, there have also been associations reported between parity and heart disease. Multiple pregnancies as well as recurrent pregnancy losses have been associated with increased risk of future ischemic heart disease [42, 43]. Interestingly, a recent cohort study demonstrated that women who successfully used fertility therapy to get pregnant did not have an increased risk of cardiovascular disease in the next 10 years, but rather there was a signal toward benefit [44].

Ovulation dysfunction has also been associated with infertility and increased risk of IHD in women. Women with polycystic ovarian syndrome (PCOS) have an increased prevalence of glucose intolerance, metabolic syndrome, and diabetes [45], which are associated with increased risk of IHD.

Women undergoing cancer treatment such as radiation or chemotherapy are at increased risk of heart disease. There is a linear relationship between the dose of ionizing radiation exposure during breast cancer radiotherapy and the risk of major coronary events in women [46]. As cancer therapy improves, this risk is of increasing importance since survivors of breast cancer are more likely to die of CVD than breast cancer. There may be ways to minimize the risk associated with radiotherapy by adjusting dosage and patient position [47•].

### Risk Prediction

Risk prediction models, such as the Framingham risk score, are largely age-dependent and only forecast 10-year risk. This can result in underestimation of the lifetime risk for women, especially young women. The AHA “Effectiveness-Based Guidelines for Prevention of Coronary Artery Disease in Women – 2011 Update” suggested a risk prediction model that relies on risk factor burden, including sex-specific risk factors, rather than age to assess a woman’s risk, and stressed the importance of taking a pregnancy history as part of the initial patient evaluation [48]. The atherosclerotic cardiovascular disease (ASCVD) risk score is a new risk assessment tool that includes stroke in the outcome. This is especially important when assessing risk in women. The calculator helps clinicians determine the need for cholesterol reducing therapy and also provides a lifetime risk assessment for patient education. The calculator incorporates the traditional risk factors used in the Framingham risk score but does not incorporate sex- specific risk factors as discussed above, so these need to be evaluated and considered separately.

## Differences in the Cardiovascular System

Although the overall coronary vasculature is similar, there are notable sex differences in the cardiovascular system. Women have smaller left anterior descending artery and right coronary artery diameters than men as assessed by computed tomography [49]. It has also been established that overall women have less atheromatous burden than men. Interestingly, this difference is most significantly seen in the coronary vascular bed and is absent in the aorta and peripheral vasculature [50]. There are also sex differences in the autonomic nervous system control of the cardiovascular system [51]. Women have more parasympathetic activity than men, who have higher sympathetic activity [52]. These differences are thought to be mediated by a variety of factors including hormonal differences [52], fat distribution [53], and psychological disorders [54]. An imbalance in the autonomic regulation in postmenopausal women has been implicated for takotsubo, a stress-mediated cardiomyopathy affecting predominantly women, as well as female-specific ischemic heart disease.

## Proposed Pathophysiology of Ischemic Heart Disease

The presumption that ischemic heart disease is the same in men and women was challenged by the Coronary Artery Surgery Study (CASS) which was a large, multicenter study that involved close to 25,000 patients of which a quarter were women. A substantial number of women referred for angiography did not have evidence of CAD, but many had positive exercise treadmill stress tests with evidence of ischemia. Based on these data, one study reported that 53 % of women vs. 12 % of men had false-positive stress tests [55], implying decreased sensitivity of the exercise treadmill test in women. However, recently, it has been suggested that angiography may be an imperfect gold standard for assessing ischemic heart disease, and these “false-positive” tests are evidence of myocardial ischemia in part of the coronary vasculature not visualized by angiography [56].

The coronary arterial system is a continuous network made of functionally distinct vessel segments of decreasing size. The initial large epicardial coronary arteries, which measure anywhere from 500 μm to 2–3 mm, are followed by the prearterioles which measure 100 to 500 μm and lead to the intramural arterioles with diameters less than 100 μm. The epicardial arteries have a primary capacitance function with minimal resistance to coronary flow. Whereas, the arterioles have a fundamental role in coronary blood flow regulation by matching myocardial oxygen demand with blood supply via changes in resistance and dilation [57].

**Fig. 1:**
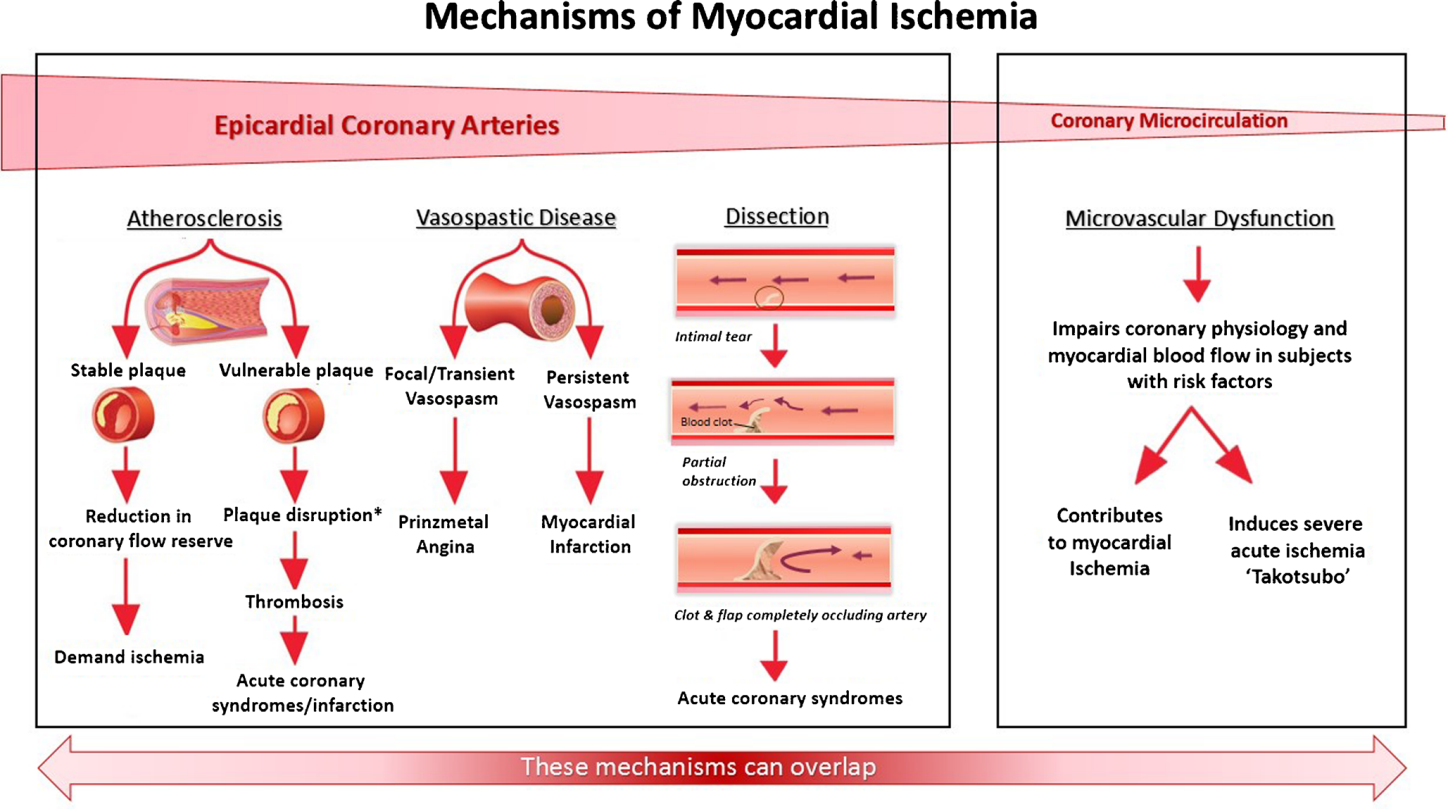
Mechanisms for ischemic heart disease in women. *Plaque disruption denotes plaque rupture or plaque erosion [60]. ** Adapted with permission from Oxford University Press and the European Society of Cardiology [105]

The underlying pathophysiology of ischemic heart disease can differ depending on the portion of the coronary vasculature affected, whether it is the large epicardial vessels or the smaller microvasculature (Fig. 1). Coronary artery disease, coronary vasospasm, and coronary artery dissection are all causes of IHD that primarily affect the epicardial coronary arteries. In contrast, microvascular dysfunction refers to dysfunction in the smaller coronary arterioles which can cause chronic ischemia, acute myocardial infarction, or stress-mediated cardiomyopathy

### Epicardial Coronary Arteries

Typically, coronary artery disease is caused by atherosclerosis in the epicardial coronary arteries. Obstruction to flow occurs either by gradual atherosclerosis accumulation or by plaque rupture resulting in occlusive thrombus formation. The utilization of coronary angiography for the diagnosis relies upon luminal obstruction resulting in contrast-void areas during angiography.

Even though a large number of women have obstructive atherosclerosis as a cause of ischemic heart disease, a substantial number of women do not. The absence of obstructive disease is identified in up to one third of women during a myocardial infarction [58] and close to two thirds of women being evaluated for ischemic chest pain [59]. Men also have nonobstructive disease on presentation, but the incidence is much lower [58].

Imaging techniques, such as intravascular ultrasound (IVUS) and optical coherence tomography (OCT) have allowed for visualization of the vessel wall and a deeper understanding of the subtle differences in the pathophysiology which were previously invisible on traditional angiography. In a prospective study of women with a myocardial infarction and nonobstructive disease on angiography (defined as <50 % stenosis in all major vessels), 29 % were noted to have evidence of plaque rupture and 12 % had plaque erosion with presumed distal embolization as evidenced by myocardial edema on MRI [60]. The term plaque disruption is a comprehensive term used to incorporate any cause of vessel wall disruption whether it is due to plaque rupture or plaque erosion. Plaque erosions are more commonly seen in women and younger individuals [61].

Coronary vasospasm has also been implicated as a cause of myocardial ischemia or injury in the absence of obstructive coronary artery disease. Although, coronary spasms usually occur in the epicardial vessels, coronary microvascular spasm can also occur [62]. Its pathogenesis is likely multifactorial involving smooth muscle hyperreactivity, endothelial dysfunction, as well as environmental factors such as smoking or alcohol consumption [63]. Focal vasospasm has been associated with atherosclerotic lesions with normal coronary flow reserve (CFR) whereas diffuse vasospasm has been associated with significantly reduced CFR suggesting a component of coronary microvascular dysfunction [63]. Coronary vasospasm is difficult to confirm on angiography and often requires provocative testing with acetylcholine or ergonovine due to the transient nature of the disease.

Lastly, spontaneous coronary artery dissection (SCAD) is an important cause of ischemic heart disease, especially in young women. In a report containing the largest series of patients with spontaneous coronary artery dissection and no atherosclerosis, the authors reported a large female predominance (82 % of the cases were women) with a 10-year major adverse cardiac event rate of up to 47 %. The LAD was the most frequent vessel affected and fibromuscular dysplasia was noted in half of the femoral angiograms done [64].

### Coronary Microvasculature

Coronary microvascular dysfunction (CMD) refers to dysfunction of the coronary arterioles measuring <500 μm in diameter which includes the prearterioles and intramuscular arterioles [57]. These arterioles are too small to be visualized by angiography and thus their function cannot be assessed by this traditional method. It is thought that microvascular dysfunction is the result of impaired relaxation or increased sensitivity to vasoconstriction which results in inappropriate myocardial blood regulation. CMD is highly prevalent in women with chest pain without obstructive disease. In fact, in the Women’s Ischemia Syndrome Evaluation (WISE) study, approximately half of the women with chest pain and ischemia without obstructive CAD had evidence of microvascular dysfunction [65]. Although microvascular dysfunction has currently been implicated as a common cause of chest pain in the absence of obstructive disease in women, whether this is a sex-specific phenomenon is still debated. Takotsubo, a stress-mediated cardiomyopathy which disproportionately affects older women, has recently been associated with microvascular dysfunction [66]. Additional evidence that microvascular dysfunction may be a problem that disproportionately affects women comes from the ARIC study where retinal artery narrowing was shown to be a marker of microvascular disease and predicted heart disease in women but not men [67]. The remaining discussion on diagnostic testing, management, and outcomes will focus on epicardial atherosclerotic disease and microvascular dysfunction as the cause of nonobstructive disease.

## Diagnostic Testing

The diagnostic testing for ischemic heart disease has historically been the same for men and women. The testing modalities can be divided into those that diagnose anatomical disease vs. those that diagnose functional ischemia. Anatomical disease can be evaluated using CT-angiography, MRI, or invasive angiography. Functional ischemia can be assessed by a variety of different stress tests as well as fractional flow reserve (FFR) measurements in the cardiac catheterization laboratory. Exercise treadmill testing has been reported as having a high false-positive rate and thus lower sensitivity in women. However, there is a shift in thinking that these may not be false-positive tests but rather evidence of ischemia due to microvascular dysfunction rather than epicardial coronary obstruction [68••]. Other prognostic tools include the Duke treadmill score which incorporates exercise time, ST-segment deviation, and angina score into an equation to help predict risk of CAD: it is a better predictor of significant CAD in women than men [69]. In addition, exercise capacity is a powerful predictor of CAD. A nomogram has been established defining age-predicted exercise capacity for women [70]. The inability to achieve 85 % of age-predicted fitness level or achieving less than 5 METs is associated with a higher risk of MI and all-cause mortality [70, 71].

The interest in evaluating microvascular dysfunction in women has brought to the forefront specific diagnostic maneuvers to assess the health of the coronary microvascular circulation. These include coronary reactivity testing (CRT) during cardiac catheterization with adenosine and acetylcholine to help diagnose coronary microvascular dysfunction and distinguish between endothelium-independent or endothelium-dependent dysfunction, respectively. The pharmacologic agents are used to induce a hyperemic state. CMD is defined as coronary volumetric blood flow increases of less than 2.5 times baseline flow during maximal hyperemic stimuli [72].

In addition, noninvasive techniques are being tested to help make the diagnosis. Women with confirmed microvascular dysfunction by CRT had decreased myocardial perfusion reserve index on cardiac MRI in response to adenosine [73]. Stress cardiac magnetic resonance (CMR) imaging is gaining interest. Women with chest pain and nonobstructive CAD who underwent adenosine CMR were found to have subendocardial ischemia more frequently compared to control subjects [74]. Lastly, positron-emission tomography (PET) can be used to assess coronary flow reserve as measured by myocardial blood flow at peak hyperemia over myocardial blood flow at baseline. The diagnostic and prognostic significance of these tests are still being explored.

When choosing a diagnostic test, there are sex-specific concerns that need to be considered such as the patient’s age and radiation exposure given the association between cumulative exposure and cancer risk in young women [68••]. The new noninvasive stress testing guidelines emphasize this concern and list expected radiation exposure for each test to help guide clinicians [68••].

## Management of Ischemic Heart Disease

### Epicardial Coronary Atherosclerosis

Even though the current treatment of coronary artery disease is similar for men and women, women are less likely to receive guideline-based therapy for the treatment of risk factors [75] and secondary prevention for known coronary artery disease [76, 77]. These include medications such as antiplatelet therapy, statins, beta blockers, and angiotensin-converting enzyme inhibitors (Table 1). In addition, after disease has been diagnosed, women are consistently referred less often to cardiac rehabilitation [78, 79] despite the known benefits. Recently, there has been increasing research into sex-related differences in the efficacy of traditional treatments. Aspirin, for unclear reasons, is more effacious for the primary prevention of myocardial infarction in men but stroke in women. Also, one study showed that guideline-based statin therapy resulted in greater atheroma regression for women than men [80].

**Table 1:** Treatment of ischemic heart disease

Epicardial coronary atherosclerosis		
Medications	Antiplatelet Agent	Recommended for all patients with CAD unless contraindicated
	ACE-I	Recommended for all patients with left ventricular ejection fraction <40 % and in those with hypertension, diabetes, or chronic kidney disease, unless contraindicated.
	Beta blockers	Therapy should be started and continued for 3 years in all patients with who have had a myocardial infarction or acute coronary syndrome
	Statin	Statin therapy should be initiated in all patients with established CAD
Lifestyle changes	BP Control	Patients with blood pressure >140/90 mmHg should be treated with lifestyle changes and medications
	Smoking Cessation	Complete cessation is recommended
	Weight Management	Goal BMI is 18.5 to 24.9 kg/m2
	Physical Activity	Recommendation is 30 min of moderate intensity activity at least 5 days a week
Other	Influenza Vaccine	Patients with cardiovascular disease should have an annual influenza vaccination.
	Cardiac Rehabilitation	Patients with the diagnosis of ACS, coronary artery bypass surgery or PCI, chronic angina and/or PAD within the past year should be referred to a cardiovascular rehabilitation program
Invasive	Possible PCI or CABG	
Vasospastic disease^a^		
Conservative	Smoking Cessation, Calcium Channel Antagonists, Long-Acting Nitrates, Magnesium, Statin	
Invasive	Possible PCI or CABG^b^	
Spontaneous coronary artery dissection^a^		
Conservative	Medical management is similar to that used in ACS and secondary prevention of epicardial coronary atherosclerosis^c^	
Invasive	Possible PCI or CABG^c^	
Microvascular dysfunction		
Conservative	Medical and lifestyle recommendations are similar to those for epicardial coronary atherosclerosis. Can also consider ranolazine for ischemic symptoms or tricyclic antidepressants for hypersensitivity to pain seen in female-specific IHD.	

*CAD* coronary artery disease, *ACE*-*I* angiotensin converting enzyme inhibitors, *BP* blood pressure, *BMI* body mass index, *ACS* acute coronary syndrome, *PCI* percutaneous coronary intervention, *PAD* peripheral artery disease, *CABG* coronary artery bypass grafting

^a^ No established guidelines

^b^ Adapted from Coronary Artery Spasm A 2009 Update [81]

^c^ Adapted from Spontaneous Coronary Artery Dissection [82]

### Coronary Vasospasm

Calcium channel blockers (CCB) are the established therapy for coronary vasospasm in addition to long-acting nitrates which can be used alone or as additive therapy to CCB (Table 1). Magnesium may have a role in acute therapy as well as prevention [81]. More invasive strategies such as percutaneous coronary intervention (PCI) and coronary artery bypass grafting are usually only for patients who are refractory to medical therapy [81].

### Coronary Artery Dissection

The treatment for coronary artery dissection remains empirical and controversial. The overall medical treatment for SCAD is similar to that recommended for patients with ACS (Table 1). However, the safety and efficacy of these medications for this specific indication have not been thoroughly evaluated. In fact, in one registry, statin therapy was associated with increased risk of recurrent SCAD [64]. This underscores the need for additional data. Traditionally, an invasive approach is taken with these patients since they often present with acute coronary syndrome; however, some are now recommending a more conservative initial approach due to the high rate of technical failure [82, 83]

### Microvascular Disease

The lack of appropriate treatment and inadequate use of secondary prevention strategies are exacerbated in patients without obstructive coronary disease [84], especially in women [85]. This is in part to the limited understanding of the disease process. In those women who are treated for female-specific ischemic heart disease, the understanding of the pathophysiology behind microvascular dysfunction has helped the development of a variety of treatment options. β-blockers improve anginal symptoms as well as functional capacity. They are more effective than channel blocker and nitrates [86] and are considered the first line of treatment. The third-generation beta blockers, nebivolol and carvedilol, also have endothelium-dependent vasodilating properties which may make them more effective than traditional β-blockers [87]. Statins and ACE-I have been shown to improve endothelial dysfunction [88, 89]. A newer agent, ranolazine, has recently been shown to improve physical functioning, angina symptoms, and quality of life in women with a positive stress test but no obstructive CAD [90]. In addition to the medications discussed, lifestyle changes such as exercise have significant benefit for symptoms of chest pain [91]. Weight loss, smoking cessation, and the Mediterranean diet all have been shown to improve endothelial function [87] and should be recommended for appropriate patients. Women with female-specific ischemic heart disease are thought to have enhanced pain sensitivity which may respond to xanthine derivatives and tricyclic antidepressants. Lastly, other nonpharmacologic treatments have shown some efficacy including cognitive-behavioral therapy, enhanced external counterpulsation, neurostimulation, and stellate ganglionectomy [87].

## Outcomes

### Epicardial Coronary Atherosclerosis

Women with coronary artery disease report worse health related quality of life outcomes compared to men [92]. In addition, there remains a pattern of higher mortality and worse cardiovascular outcomes in women with ischemic heart disease [93, 94]. This is partially attributed to the incomplete use of secondary prevention treatment regimens for coronary atherosclerosis which improve survival, reduce recurrent ischemic events, and improve quality of life [95].

There are also sex differences in invasive strategies during an ACS presentation. Compared with men, women are at increased risk of adverse outcomes after acute coronary syndrome as well as percutaneous coronary interventions [96, 97]. They are also at increased risk of bleeding from medical therapies used in acute coronary syndrome and the use of femoral access for PCI [98, 99].

### Microvascular Dysfunction

It was originally thought that women with nonobstructive disease had a benign prognosis [100]; however, we now know that this is not the case. Some of the discrepancies in findings can be attributed to patient selection and the heterogeneity of inclusion criteria in studies. Women with proven ischemia but no obstructive coronary artery disease still have increased events compared to asymptomatic women [101]. In fact, women with stable angina and nonobstructive CAD are three times more likely than men to experience a cardiac event within the first year of cardiac catheterization [102]. In those with ACS and nonobstructive disease, there is a 2 % risk of death and MI at 30 days [103]. Within the WISE Study, a subset of women without angiographic CAD but persistent symptoms underwent magnetic resonance spectroscopy (MRS) to assess for myocardial ischemia. Those women with no obstructive CAD and a normal MRS had a 13 % vascular event rate in the following 3 years; whereas those with an abnormal MRS study had a cardiovascular event rate of 43 % which was similar to the reference WISE women with obstructive CAD (48 %) [104]. More research is needed to determine the best diagnostic testing and treatment strategies for these women.

## Conclusion/Future Directions

We have come a long way in the past 25 years since the US National Institutes of Health (NIH) mandated the inclusion of women in all NIH-sponsored research in 1990. However, we are only beginning to scratch the surface in the understanding of sex differences in the pathophysiology and treatment of ischemic heart disease. There are important differences that clinicians should be cognizant of as discussed in this article. These differences impact our understanding of the disease, diagnosis, and treatment. In the future, as we continue to reevaluate our known therapies based on sex, we will be able to adjust our treatment strategies accordingly. There remain many biologic, pathophysiologic, and diagnostic sex differences in ischemic heart disease that have yet to be clarified and will require additional research.
